# Selective digestive decontamination with oral colistin plus gentamicin for persistent bacteraemia caused by non-carbapenemase-producing carbapenem-resistant *Klebsiella pneumoniae* in a neutropenic patient

**DOI:** 10.1093/jacamr/dlab079

**Published:** 2021-06-21

**Authors:** Maria Spencer-Sandino, Roberto Riquelme-Neira, William C Shropshire, An Q Dinh, Gerardo González-Rocha, Paulina González-Muñoz, Alejandra Vera-Leiva, Rafael Araos, Blake Hanson, Cesar A Arias, José M Munita

**Affiliations:** Genomics and Resistance Microbes (GeRM) Lab, Facultad de Medicina CAS-UDD, Instituto de Ciencias e Innovación en Medicina (ICIM), Santiago, Chile; Millennium Initiative for Collaborative Research on Bacterial Resistance (MICROB-R), Santiago, Chile; Genomics and Resistance Microbes (GeRM) Lab, Facultad de Medicina CAS-UDD, Instituto de Ciencias e Innovación en Medicina (ICIM), Santiago, Chile; Millennium Initiative for Collaborative Research on Bacterial Resistance (MICROB-R), Santiago, Chile; Center for Infectious Diseases, School of Public Health, University of Texas Health Science Center, Houston, TX, USA; Center for Antimicrobial Resistance and Microbial Genomics, Division of Infectious Diseases, University of Texas McGovern Medical School at Houston, Houston, TX, USA; Center for Infectious Diseases, School of Public Health, University of Texas Health Science Center, Houston, TX, USA; Center for Antimicrobial Resistance and Microbial Genomics, Division of Infectious Diseases, University of Texas McGovern Medical School at Houston, Houston, TX, USA; Millennium Initiative for Collaborative Research on Bacterial Resistance (MICROB-R), Santiago, Chile; Universidad de Concepción, Facultad de Ciencias Biológicas, Departamento de Microbiología, Laboratorio de Investigación en Agentes Antibacterianos, Concepción, Chile; Millennium Initiative for Collaborative Research on Bacterial Resistance (MICROB-R), Santiago, Chile; Universidad de Concepción, Facultad de Ciencias Biológicas, Departamento de Microbiología, Laboratorio de Investigación en Agentes Antibacterianos, Concepción, Chile; Universidad de Concepción, Facultad de Ciencias Biológicas, Departamento de Microbiología, Laboratorio de Investigación en Agentes Antibacterianos, Concepción, Chile; Genomics and Resistance Microbes (GeRM) Lab, Facultad de Medicina CAS-UDD, Instituto de Ciencias e Innovación en Medicina (ICIM), Santiago, Chile; Millennium Initiative for Collaborative Research on Bacterial Resistance (MICROB-R), Santiago, Chile; Center for Infectious Diseases, School of Public Health, University of Texas Health Science Center, Houston, TX, USA; Center for Antimicrobial Resistance and Microbial Genomics, Division of Infectious Diseases, University of Texas McGovern Medical School at Houston, Houston, TX, USA; Center for Infectious Diseases, School of Public Health, University of Texas Health Science Center, Houston, TX, USA; Center for Antimicrobial Resistance and Microbial Genomics, Division of Infectious Diseases, University of Texas McGovern Medical School at Houston, Houston, TX, USA; Department of Microbiology and Molecular Genetics, University of Texas McGovern Medical School at Houston, Houston, TX, USA; Molecular Genetics and Antimicrobial Resistance Unit-International Center for Microbial Genomics, Universidad El Bosque, Bogota, Colombia; Genomics and Resistance Microbes (GeRM) Lab, Facultad de Medicina CAS-UDD, Instituto de Ciencias e Innovación en Medicina (ICIM), Santiago, Chile; Millennium Initiative for Collaborative Research on Bacterial Resistance (MICROB-R), Santiago, Chile; Department of Microbiology and Molecular Genetics, University of Texas McGovern Medical School at Houston, Houston, TX, USA

## Abstract

**Background:**

Carbapenem-resistant *Klebsiella pneumoniae* (CR*Kp*) have become an increasing public health problem worldwide. While most CR*Kp* around the world harbour a carbapenemase enzyme, the clinical relevance of non-carbapenemase-producing CR*Kp* (non-CP-CR*Kp*) is increasingly recognized. Selective digestive decontamination (SDD) has been proven successful as a decolonization strategy for patients colonized with Gram-negatives in the ICU. However, it is not regularly used to treat invasive infections.

**Objectives:**

To report the use of SDD as a useful strategy for managing recalcitrant CR*Kp* bloodstream infections.

**Patients and methods:**

We present a neutropenic patient with a recalcitrant bloodstream infection with non-CP-CR*Kp* treated with SDD. Besides, genomic analyses of five isolates of non-CP-CR*Kp* was performed.

**Results:**

After 11 days of SDD treatment with oral colistin and gentamicin, bacteraemia was successfully eradicated. Genomic analysis indicates a fully carbapenem-resistant phenotype evolved *in vivo* and suggests that the mechanism of carbapenem resistance in our strains relates to gene amplification of narrow-spectrum β-lactamases.

**Conclusions:**

Our report highlights that SDD might be a useful strategy to manage CR*Kp* bloodstream infections, when intestinal translocation is the likely source of the bacteraemia. In addition, the development of a resistant phenotype during therapy is worrisome as therapies directed against these organisms are likely to favour the amplification process.

## Introduction

Carbapenem-resistant Enterobacterales (CRE) have become a major public health problem worldwide,^1^ with carbapenem-resistant *Klebsiella pneumoniae* (CR*Kp*) being responsible for most CRE infections in clinical practice.^2^ Although most CR*Kp* around the world harbour a carbapenemase enzyme able to degrade carbapenems,^3^ the clinical relevance of non-carbapenemase-producing CR*Kp* (non-CP-CR*Kp*) is increasingly recognized.^4^ A recent study reported similar outcomes among patients infected with carbapenemase-producing and non-CP-CR*Kp*.^5^ The mechanisms leading to carbapenem resistance in non-CP-CR*Kp* are not fully known. It has been associated with the presence of ESBL and disruption of porin channels.^1^ Additionally, *in vivo* gene amplification of narrow-spectrum β-lactamases during carbapenem therapy was recently postulated as a mechanism of carbapenem resistance in non-carbapenemase-producing CRE.^4^

Selective digestive decontamination (SDD) with non-absorbable, enterally administered antibiotics (e.g. tobramycin, colistin), has been suggested as a suitable decolonization strategy for patients colonized with CR*Kp*^6^ and to prevent infections due to aerobic Gram-negative bacilli in critically ill patients.^7^ SDD is not routinely used as a therapeutic strategy for invasive bacterial infections. We report a neutropenic patient with relapsing/refractory AML, hospitalized in Chile, with persistent non-CP-CR*Kp* bacteraemia and whose bloodstream was ultimately sterilized after SDD with a combination of oral colistin and gentamicin.

## Patient and methods

### Ethics

The patient provided written informed consent before this clinical case was reported (version 1.1, 28/11/2013). This study was approved by the institutional review board: Comité Ético Científico, Facultad de Medicina Clínica Alemana, Universidad del Desarrollo, Santiago, Chile (IRB numbers 2018-54 and 2021-46).

### Case report

**Figure 1.**
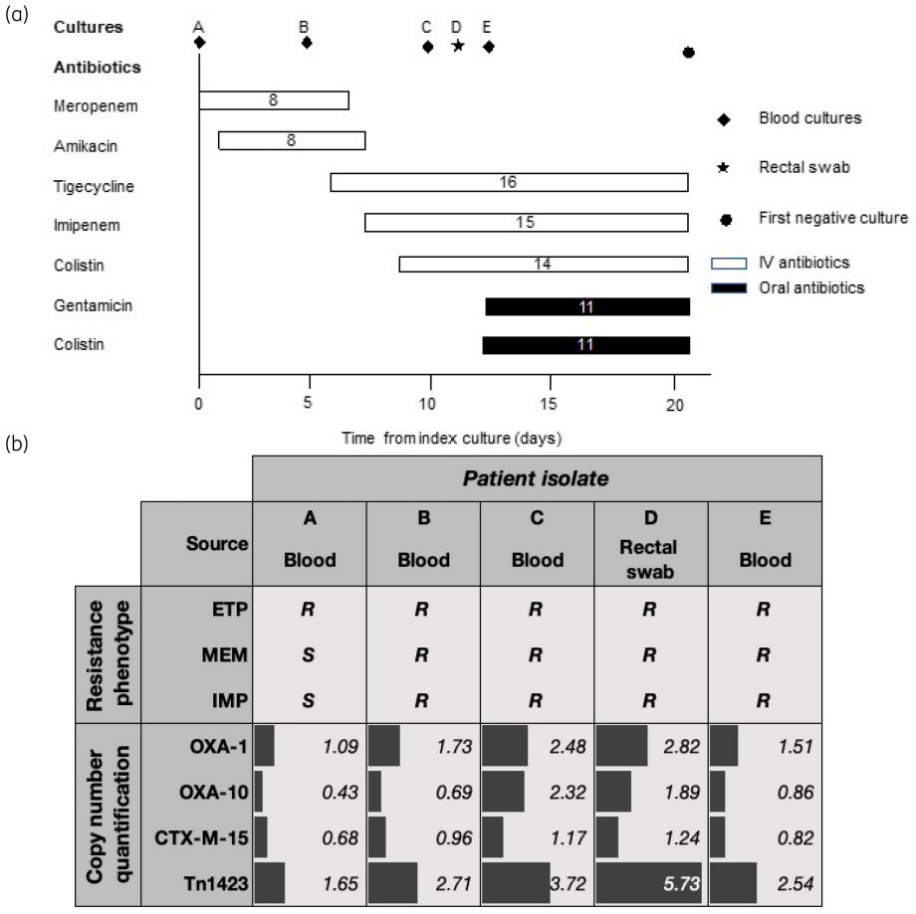
(a) Treatment timeline of the patient and *K. pneumoniae* isolates by body site. Uppercase letters (A to E) represent cultures positive for CR*Kp*; numbers in boxes represent days of treatment with the antibiotic listed to the left; diamonds, blood cultures; star, rectal swab; circle, first negative blood culture and end of SDD. (b) Summary table of the patient’s CR*Kp* isolates versus carbapenem resistance phenotype (R or S); copy number quantification expressed in estimated copy number. Uppercase letters (A to E) represent cultures positive for CR*Kp*. ETP, ertapenem; MEM, meropenem; IMP, imipenem; R, resistant; S, susceptible; Tn*1423*, IS*26*-mediated translocatable element.

A 27-year-old man with relapsing/refractory AML presented with fever (101.8 °F) and diarrhoea, 9 days after receiving his second cycle of fludarabine, cytarabine, idarubicin and G-CSF (FLAG-IDA). Initial lab work revealed profound neutropenia (absolute neutrophil count [ANC] of 0 cells/mm^3^). Cultures were drawn and he was empirically started on IV meropenem (1 g q8h). Blood cultures revealed MDR *K. pneumoniae*, resistant to ertapenem and susceptible to imipenem, meropenem, amikacin and colistin (MICs of 1, 2, 16 and 2 mg/L, respectively) (Table S1, available as Supplementary data at *JAC-AMR* Online). Meropenem was increased to 2 g q8h and IV amikacin 1 g q24h was added. The patient continued persistently febrile and neutropenic (ANC = 0 cells/mm^3^). CT scan of the lung was unremarkable, and imaging of the abdomen revealed enterocolitis. Follow-up blood cultures at Day 5 of therapy remained positive for a CR*Kp*, which was now fully resistant to all carbapenems (Table S1, Figure 1b). A central line was removed, and therapy was switched to IV imipenem 1 g q6h (due to lower MICs, see Table S1) plus tigecycline (100 mg loading dose; 50 mg q12h thereafter) and colistin (300 mg loading dose; 150 mg q8h thereafter). The patient remained febrile, hemodynamically stable, neutropenic and with diarrhoea. A film array of the stools was negative and blood cultures at Day 9 of bacteraemia remained positive for MDR CR*Kp* with similar susceptibilities (Table S1). A swab obtained from a fresh faecal sample at Day 11 after the index culture yielded a CR*Kp* with the same antimicrobial susceptibility as those recovered from the bloodstream (Table S1). Intestinal translocation was postulated as the presumed source of the bacteraemia as no other foci were clinically apparent. After discussion with the patient, we made the decision to start SDD with oral colistin (100 mg q6h) plus gentamicin (80 mg q6h). Systemic antimicrobials were continued without changes (Figure 1a). After 4 days of SDD plus IV antimicrobials, the patient became afebrile for the first time in the hospital course and follow-up blood cultures obtained that same day were negative (Figure 1a), despite continuous neutropenia (ANC = 0 cells/mm^3^). The patient completed 11 days of the latter regimen, remaining afebrile. His neutropenia recovered 17 days after bacteraemia clearance and was discharged home on Day 32 of hospitalization.

### Bacterial isolates, identification and susceptibility testing

Five isolates of *K. pneumoniae* were analysed, four recovered from blood cultures and one from a swab obtained from a fresh faecal sample. Isolates were identified by MALDI-TOF. Susceptibilities were determined by VITEK 2 system (bioMérieux, France), except for colistin and carbapenems (ertapenem, meropenem and imipenem), which were performed by broth microdilution (BMD).^8^

### Genome sequencing and bioinformatic analysis

Genomic DNA from all isolates was extracted using DNeasy Blood and Tissue kit (QIAGEN, Germany), following manufacturer's recommendations. Library preparation was performed using NexteraXT (Illumina, USA) and the Illumina MiSeq platform was used for short-read sequencing. Bioinformatic analyses were performed using a previously reported pipeline.^4^*In silico* MLST was determined by mlst-v2.15.1 (see Supplementary data). AMR characterization was performed using ABRIcate with the Comprehensive Antimicrobial Resistance Database (CARD).

ONT was used to perform long-read sequencing using the GridION X5 platform (Oxford, UK). The Rapid Barcoding Kit (SQK-RBK004) was used for library preparation for sequencing on the R9.4.1 flow-cell. Guppy-v3.2.2 (Oxford, UK) was used for base-calling and read filtering. A previously published consensus ONT pipeline was used for generating genome assemblies.^4^

We were able to estimate copy number variants using a pipeline that normalizes coverage depth of genes of interest to single copy essential genes. Used the pubMLST schema for *K. pneumoniae* ST25 to normalize coverage depth to genes of interest (e.g. *bla*_OXA-10_, *bla*_CTX-M-15_) by short- and long-read alignment using bwa, minimap2, SAMtools and bcftools.^9^^,^^10^

### Data availability

Illumina short-read data are available for Isolates A–E (Table S2) in the NCBI repository BioProject PRJNA664089. ONT long-reads as well as the consensus assembly for the index isolate (Isolate A; sample Name 1423) are publicly available (GenBank no. GCA_014788765.1).

### Outer membrane protein analysis

Porin profiles were determined by SDS-PAGE using 12% polyacrylamide gels as previously reported.^11^

## Results

### Genomic analysis

Short-read WGS analysis demonstrated all five isolates belonged to the ST25 lineage and harboured an identical resistome profile (Tables S1 and S3) with serial strains having less than three pairwise SNP differences compared with the index strain. All genomes showed the presence of the ESBL *bla*_CTX-M-15_ as well as the narrow spectrum β-lactamases *bla*_OXA-1_ and *bla*_OXA-10_ (Figure 1b). Carbapenemases were not detected in any of the isolates. Porin analyses revealed a predicted truncation of *ompK35* due to an insertion sequence IS1R in all isolates (Table S1); *ompK36* and *ompK37* were intact. SDS-PAGE confirmed the absence of OmpK35 and presence of OmpK 36/37 in all isolates (Figure S1).

Copy number quantification using the combined short-read and Oxford Nanopore Technologies (ONT) assemblies suggested a significant increase in the copy number of *bla*_OXA-1_ and *bla*_OXA-10_ (Figure 1b and Table S2). The increase in gene copy number was associated with the rise in MICs of all carbapenems (Table S1). Further analysis revealed the presence of an IS*26*-mediated translocatable element recombined with Tn*5403*. This transposon, designated Tn*1423*, was associated with carriage of *bla*_OXA-1_ on both FII_k_/FIB_k_ (GenBank no. CP061833) and R-type (GenBank no. CP061834) plasmids. Amplification of the Tn*1423* unit was detected in all isolates as compared with the index culture. Following SDD, a decreasing trend in the copy number of *bla*_CTX-M-15_ and Tn*1423* harbouring *bla*_OXA-1_ was observed (Figure 1b and Table S2).

## Discussion

Infections due to CR*Kp* pose a major clinical challenge due to the scarcity of therapeutic options. This is particularly relevant in countries where access to recently approved antibiotics targeting CR*Kp* is limited. Indeed, ceftazidime/avibactam, meropenem/vaborbactam or imipenem/relebactam were not available in Chile at the time of the patient’s presentation. Thus, faced with no reliable therapeutic alternatives, in a severely immunocompromised patient with persistent CR*Kp* bacteraemia for >10 days, we decided to use SDD under the premise that intestinal translocation was a major source of bacteraemia in neutropenic patients.^12^

Subjects with haematological malignancies are particularly prone to invasive bacterial infections, due to a combination of risk factors such as prolonged neutropenia, frequent hospitalizations and exposure to antimicrobials, among others.^12^ SDD has been successfully used for prophylaxis of infections in the ICU, with previous studies demonstrating a decrease in healthcare-associated infection and mortality.^6^^,^^12^ SDD with oral gentamicin proved efficacious to achieve intestinal eradication in patients colonized with CR*Kp* undergoing stem cell transplantation.^7^^,^^12^ However, data to support the use of SDD to manage active MDR infections are scant. A previous report used oral bacitracin to help treat a recalcitrant bacteraemia due to VRE in a leukaemia patient,^13^ suggesting that this approach may help in situations where systemic antibiotic therapy fails, as in our case. Previous data on patients undergoing renal replacement therapy receiving SDD with tobramycin suggested the possibility of intestinal absorption of the antibiotic.^12^ Although our patient had a normal renal function, the possibility of partial intestinal absorption contributing to the clearance of the bacteraemia cannot be ruled out. The ‘collateral’ impact of this strategy in the gut microbiota is unknown. It is likely that our patient’s microbiota was dominated by CR*Kp* and decreasing numbers of these organisms to an appropriate ‘threshold’ likely stopped translocation to the bloodstream.^14^ With the availability of microbiome analyses, identifying patients at risk of persistent bacteraemia due to intestinal domination with certain MDR organisms may open up the possibility of using SDD in specific cases, as a targeted intervention.

Non-CP-CR*Kp* are increasingly recognized as a public health concern. A recent US-based prospective cohort study demonstrated 41% of all CRE isolates did not carry carbapenemases.^5^ Although the mechanisms of carbapenem resistance in non-CP-CR*Kp* are not fully understood, our genomic analyses, along with recently data,^4^ suggest that the isolates from our patient developed carbapenem resistance after amplification of *bla*_OXA-1_ and *bla*_OXA-10_ (narrow-spectrum β-lactamases) and concomitant *ompK35* disruption. The development of this phenotype during therapy is worrisome as therapies directed against these organisms are likely to favour the amplification process.

In summary, our report highlights that SDD might be a useful strategy to manage recalcitrant CR*Kp* bloodstream infections, particularly when intestinal translocation is the likely source of the bacteraemia.

## Funding

This work was supported by FONDECYT (1211947 to J.M.M.); ANID Millennium Science Initiative/Millennium Initiative for Collaborative Research on Bacterial Resistance (MICROB-R), Government of Chile (NCN17_081 to J.M.M., R.A., G.G.-R. and P.G.-M.); and Departamento Cientifico Docente, Clinica Alemana de Santiago (to J.M.M.). C.A.A. is also supported by NIH/NIAID grants R01 AI134637, R01 AI148342 and K24 AI121296, UTHealth Presidential Award and University of Texas System STARS Award.

## Transparency declarations

J.M.M. has received unrestricted research grants from Pfizer, MSD and bioMérieux. C.A.A. has received grant support from Merck, MeMed Diagnostics and Entasis Therapeutics. All other authors: none to declare.

## Supplementary data

Tables S1 to S3, Bioinformatics and Figure S1 are available as Supplementary data at *JAC-AMR* Online.

## Supplementary Material

dlab079_Supplementary_Data
