# Supplementary material for: Selective digestive decontamination with oral colistin plus gentamicin for persistent bacteraemia caused by non-carbapenemase-producing carbapenem-resistant Klebsiella pneumoniae in a neutropenic patient
Source: JAC Antimicrob Resist. 2021 Jun 21;3(2):dlab079. doi: 10.1093/jacamr/dlab079 (PMC8215431; doi:10.1093/jacamr/dlab079)
Supplement: dlab079_Supplementary_Data [file dlab079_supplementary_data.zip › Supplementary_data.docx]

**Supplementary data**


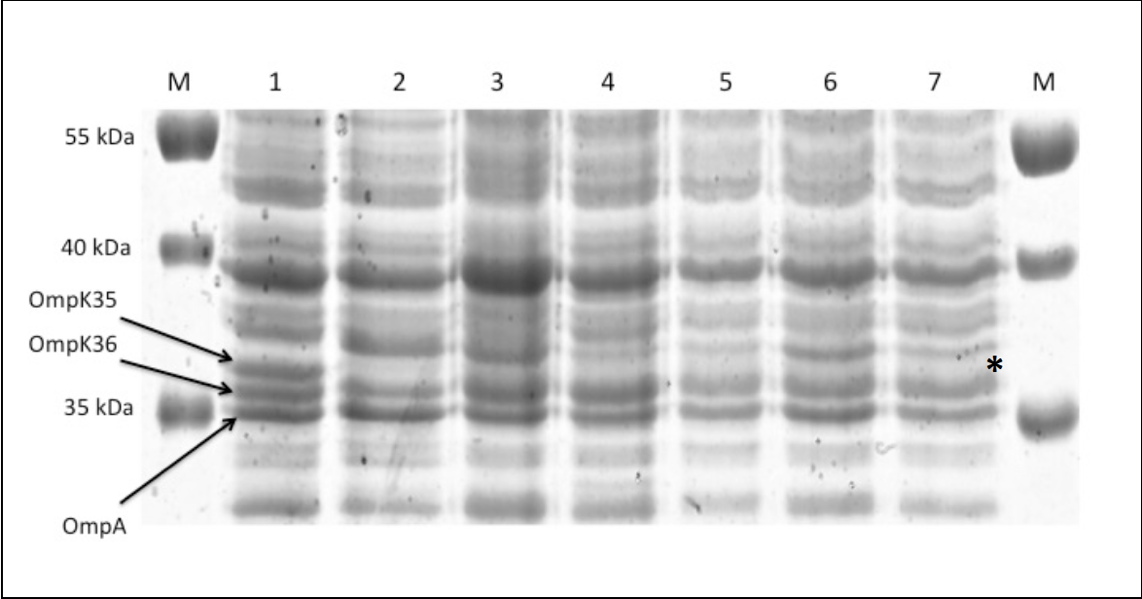


**Figure S1**. SDS-PAGE outer membrane proteins profile of all 5 isolates (A - E). M, molecular marker; 1, Control isolate *K*. *pneumoniae* 50 (OmpK35 [+]); 2, Control isolate *K*. *pneumoniae* UCO338 (OmpK35 [-]). The asterisk depicts the absence of the OmpK35 band.

**Supplementary bioinformatics**

Seemann T, mlst Github: <https://github.com/tseemann/mlst>

Seemann T, Abricate Github: https://github.com/tseemann/abricate

https://card.mcmaster.ca/

nanoporetech, GitHub: https://github.com/nanoporetech/qcat

Shropshire W, flye_hybrid_assembly_pipeline GitHub: https://github.com/wshropshire/flye_hybrid_assembly_pipeline

Shropshire W, convict GitHub: https://github.com/wshropshire/convict
